# Functional morphology, biomechanics and biomimetic potential of stem–branch connections in *Dracaena reflexa* and *Freycinetia insignis*

**DOI:** 10.3762/bjnano.2.21

**Published:** 2011-03-24

**Authors:** Tom Masselter, Sandra Eckert, Thomas Speck

**Affiliations:** 1 Plant Biomechanics Group Freiburg, Botanic Garden, Faculty of Biology, University of Freiburg, Schänzlestraße 1, 79104 Freiburg, Germany; 2 Competence Network Biomimetics and Bionics Competence Network BIOKON e.V

**Keywords:** Biomimetics, branching, *Dracaena reflexa*, *Freycinetia insignis*, monocotyledons

## Abstract

Branching in plants is one of the most important assets for developing large arborescent growth forms with complex crowns. While the form and development of branching in gymnosperms and dicotyledonous trees is widely understood, very little is known about branching patterns and the structure of branch–stem-junctions in arborescent monocotyledons. For a better and quantitative understanding of the functional morphology of branch–stem-junctions in arborescent monocotyledons, we investigated the two species *Dracaena reflexa* and *Freycinetia insignis*. While *D. reflexa* is able to develop large arborescent forms with conspicuous crowns by anomalous secondary growth, *F. insignis* remains relatively small and is only capable of primary growth. Biomechanical investigations were performed by applying vertical loads up to rupture to lateral branches of both species. This allows the analysis of the fracture mechanics and the determination of the maximal force, stress and strain at rupture as well as the fracture toughness. Functional morphology was correlated with the mechanical behaviour of these plants and compared to data of other dicotyledonous trees. The high energy absorption found in the rupture process of lateral branches of *D. reflexa* and *F. insignis* makes them promising biological concept generators with a high potential for biomimetic implementation, i.e., for the development of branched fibre-reinforced technical composites. A wide range of constructional elements with branched (sub-)structures can be optimised by using solutions inspired by plant ramifications, e.g., in automotive and aerospace engineering, architecture, sports equipment and prosthetic manufacturing.

## Introduction

One of the most conspicuous features of woody plants is their ability to form branches and canopies. Some of these branches can grow continuously and be as long-lived as the stems. This has always intrigued naturalists and botanists, and therefore, branching in woody plants has been the subject of scientific studies for centuries. These studies have increased in number over the past decades and improved our understanding of the importance and the form–structure–function-relationship of branching significantly (e.g., [1–9]). It has become evident that branching is essential for woody plants, as it allows space to be occupied and trap solar energy in an efficient way (e.g., [5]). However, these benefits are coupled with disadvantages such as increased magnitude and complexity of mechanical loads. Therefore, structural and mechanical adaptation on different hierarchical levels can be observed both in main stems as well as in lateral branches [9–12], and particularly in the region of branch–stem-junctions [4,6–8]. Due to these optimisations of form and structure, notch stresses in branch–stem-junctions can be significantly reduced or even avoided [6–8]. These ramifications are often highly stiff yet flexible enough to avoid fracture or tearing apart even under high mechanical loading.

**Figure 1:**
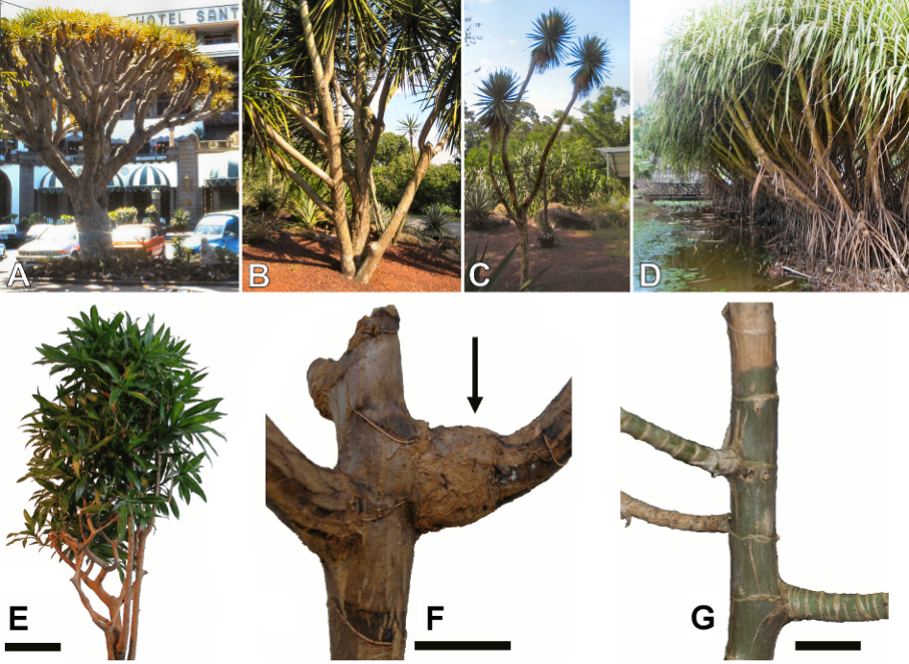
Arborescent monocotyledons. (A) *Dracaena draco*, (B) *Dracaena yuccaeifolia,* (C) *Yucca* sp.*,* (D) *Pandanus* sp. © Thomas Speck. Morphology of monocotyledons analysed. (E) Branched specimen of *Dracaena reflexa.* Scale bar = 200 mm, (F) Detail of a branched specimen of *Dracaena reflexa*. Anomalous secondary growth has led to a thickened zone in the region of branch–stem-junction (arrow). Scale bar = 20 mm, (G) Branched specimen of *Freycinetia insignis.* Scale bar = 15 mm.

**Figure 2:**
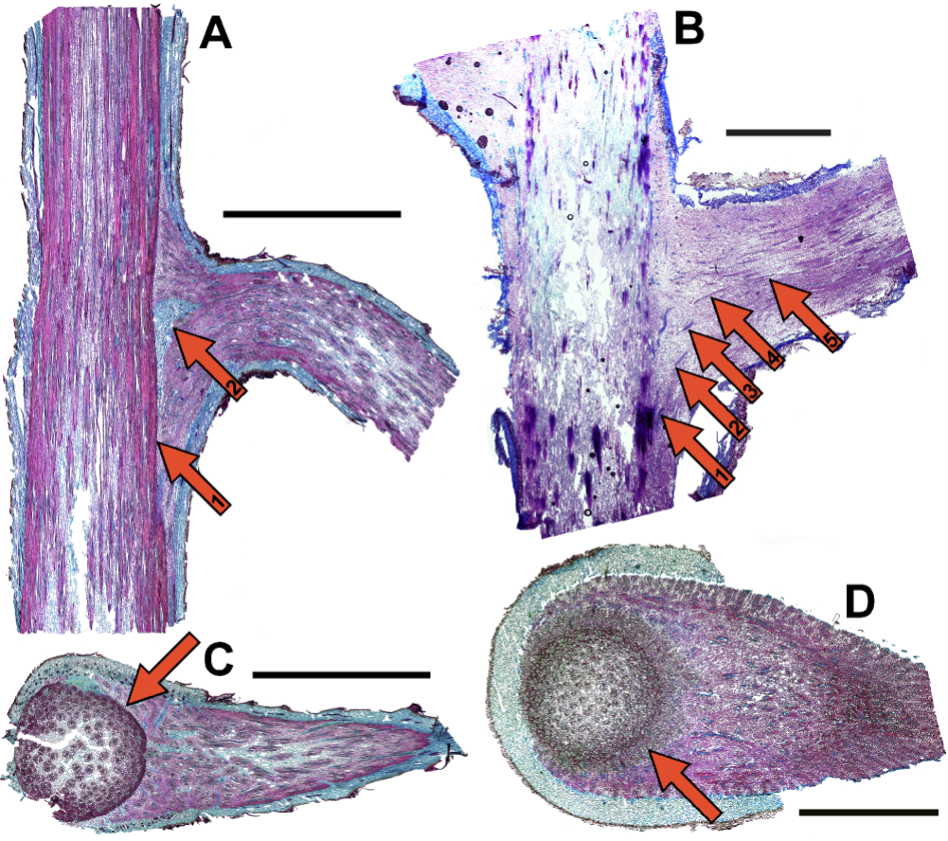
Branch–stem-junction of *Freycinetia insignis* and *Dracaena reflexa*. (A) *F. insignis*, longitudinal section, arrow 1: distinct border-line between fibrous bundles in the main stem and in the lateral branch, arrow 2: zone of partly lignified parenchymatous ground tissue without fibrous bundles, (B) *D. reflexa*, longitudinal section, arrows 1–5; gradual transition from fibrous bundles in the main stem (arrow 1) to fibrous bundles in the lateral branch (arrow 5), (C) *F. insignis*, cross-section, arrow: distinct border-line between fibrous bundles in the main stem and in the lateral branch, (D) *D. reflexa*, cross-section, arrow: gradual transition zone showing fibrous bundles in the main stem and fibrous bundles in the lateral branch. Scale bars = 3 mm.

While numerous studies exist on branching in gymnosperms and dicotyledonous trees, relatively little is known about branching in monocotyledons. This could be due to the fact that branching is observed much more frequently in broad leaved and gymnosperm trees with secondary cambial growth than in arborescent monocotyledons. Monocotyledons seldom branch, normally only after bloom with a terminal inflorescence or when the apical meristem of the axis is damaged or destroyed [13]. The ramifications of monocotyledons can vary considerably in shape and number (Figure 1). The angle between the main stem and the lateral branch can differ considerably among monocotyledons. Small monocotyledons such as *Freycinetia* often show high values for stem–branch angles of up to 90° (Figures 1G, 2B), which are comparable to the values found in many gymnosperms and broad leaved trees. Other monocotyledons, such as species of *Dracaena* or *Yucca*, can grow much higher, developing considerable stem dimensions. They develop large arborescent growth forms often with crowns (Figure 1) due to their ability to increase stem and branch diameter by anomalous secondary growth. Thus, additional fibrous bundles in an often lignified parenchymatous ground tissue are differentiated due to secondary growth processes by a cambium located in the cortex region [3,14–19]. In these monocotyledons, the stem–branch angles typically are much lower, about 60°, but can vary considerably (Figure 1 and Figure 2, see [1]). However, the degree of the branching angles is to some extent limited by the inner bauplan of these plants. In contrast to gymnosperms and dicotyledonous, trees in which the wooden tissues are compact and a relatively large amount of secondary wood from the main stem is in direct connection with the branches, in arborescent monocotyledons vascular bundles with fibre caps (both summed up as ‘fibrous bundles’ in this study) are isolated, i.e., with no or little tangential or radial interconnection, and arranged in a parenchymatous, often lignified cellular ground tissue [14,17–25] (Figure 2). This makes the stems flexible, yet decreases their static load-bearing capacity. It can be speculated that branching angles are limited in large arborescent monocotyledons so that critical bending moments leading to fracture are avoided.

Some of the fibrous bundles run from the main stem into the lateral branches [2]. Apart from that, very little is known about their anatomy. The arrangement and course of the fibrous bundles in branch–stem-junctions of arborescent monocotyledons and the functional morphology and mechanics of branch–stem-junctions have yet not been analysed quantitatively. For our studies we chose two branched arborescent monocotyledons, *Dracaena reflexa* and *Freycinetia insignis*, in order to answer the following questions:

1. How are the fibrous bundles in the main stem connected to those in the lateral branch? and what are the structure and arrangement of the fibrous bundles in the regions of branch–stem-junctions?
2. What are the biomechanical properties of the branch–stem-junctions?
3. Does a quantifiable relationship exist between morphology/anatomy and the biomechanical properties of the branch–stem-junctions?
4. Are the values of the biomechanical properties (i.e., maximal force, fracture toughness as well as stress and strain) of the branch–stem-junctions in arborescent monocotyledons comparable to the values found in dicotyledonous trees?

## Results

### Morphology

In *Dracaena reflexa*, the fibrous bundles of the lateral branches clasp around the main stem resulting in a flange mounted structure (Figure 2B,D, see Supporting Information File 1). Some of the largely paraxial fibrous bundles in the main stem are connected with the predominantly fibrous bundles in the lateral branches so that there is no distinct separation boundary between the bundles in the stem and the bundles in the branch (Figure 2B,D) but rather a gradual transition. The branch bundles are also paraxial oriented with regard to the lateral branch resulting in a perpendicular orientation relative to the main stem (Figure 2B,D, see Supporting Information File 1). In *F. insignis*, the fibrous bundles are arranged in a generally similar pattern that, however, differs in several important aspects. The fibrous bundles of the lateral branches also clasp around the stem. However, the fibrous bundles of the lateral branches are not directly interconnected with the fibrous bundle system in the median section of the main stem (Figure 2A,C). Additionally, it is noticeable that there exists a purely parenchymatous region between the main stem and the lateral branches in the region of the branch–stem-junction (Figure 2A).

### Biomechanical tests

**Figure 3:**
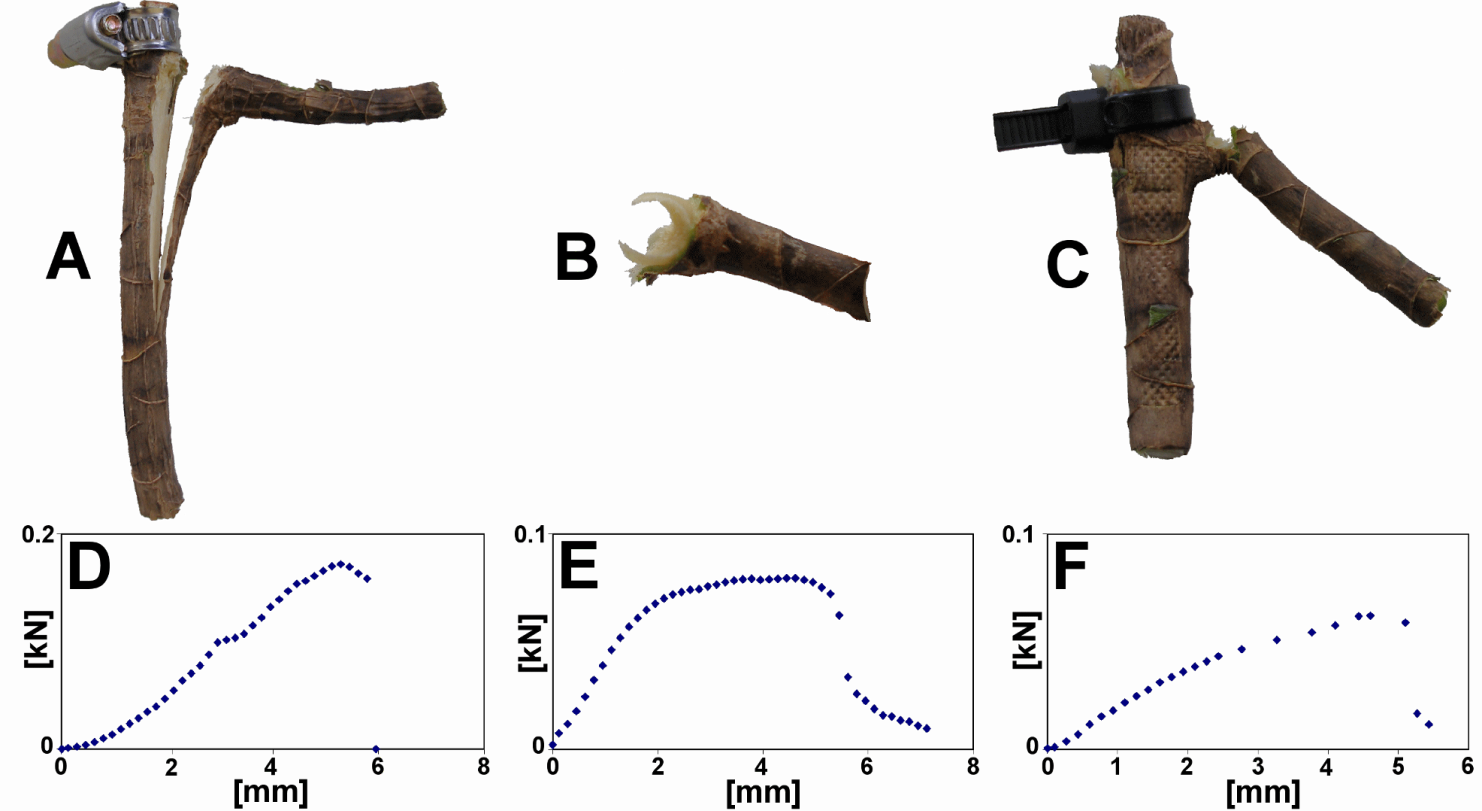
Breaking experiments. Different modes of fracture found in *Dracaena reflexa*. (A) Fracture in the stem, (B) sickle-shaped detachment, (C) fracture in the branch; (D, E, F) force-displacement-curves resulting from the experiments carried out in A, B, and C, respectively.

Three main modes of mechanical failure (Figure 3) were found in the 31 samples of *Dracaena reflexa* when the branches were loaded with over-critical forces: (1) failure in the stem (11 samples, Figure 3A) with delamination between the fibrous bundles and the parenchymatous ground tissue resulting in longitudinal splitting of the main stem, (2) sickle-shaped detachment of the lateral branch (13 samples, Figure 3B), i.e., failure in the region where the branch is connected to the stem, and (3) failure in the branch (7 samples, Figure 3C). The different modes of failure are correlated with typical shapes of the force-displacement-curves (Figure 3D–F).

Two modes of failure occurred in the 19 samples of *Freycinetia insignis*: (1) Most often a detachment of the branch resulting in a flat fracture surface was observed (16 samples), while (2) failure in the region where the branch is connected to the stem occurred in three cases only.

#### Maximal force *F*_max_ [kN]

**Figure 4:**
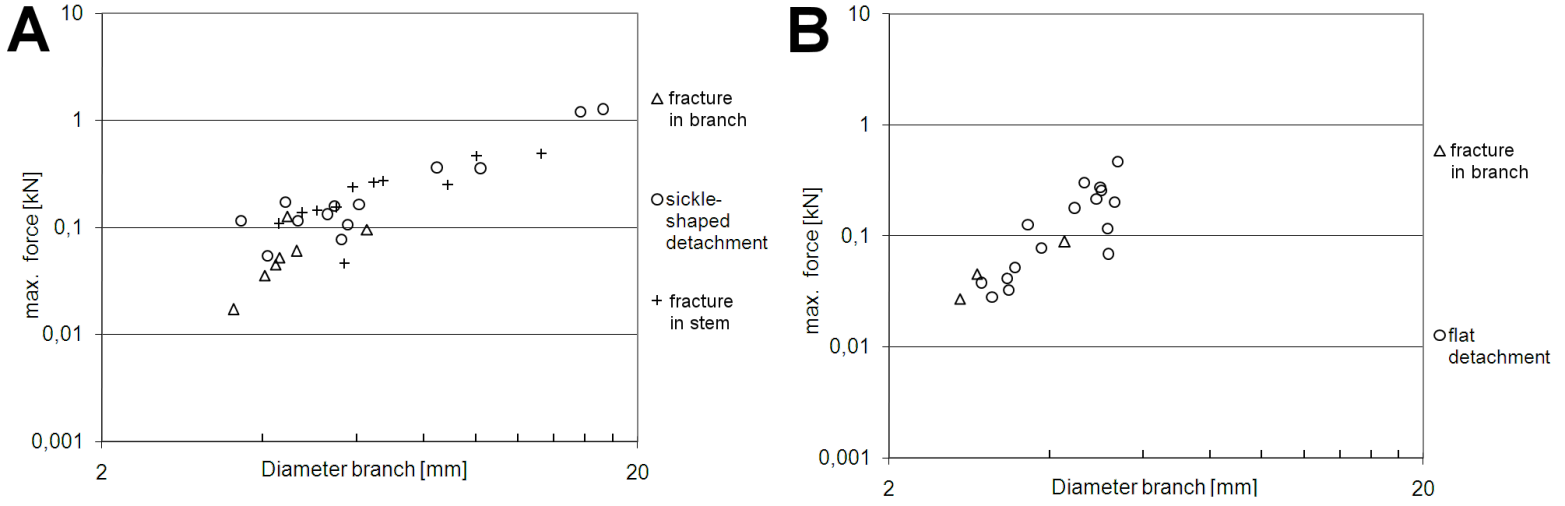
Bivariate, double logarithmic plot of maximal forces vs diameter of lateral branches (*d*_1_ or *d*_2_ depending on the mode of fracture (see ‘Experimental’ section ) in *Dracaena reflexa* (A) and *Freycinetia insignis* (B).

The maximal force *F*_max_ increases with increasing diameter of the lateral branches (Figure 4, Table 1). This correlation is statistically significant. It holds true for each mode of failure and the pooled data of all fracture modes in *Dracaena reflexa* (Figure 4A), and for the mode of flat detachment in *Freycinetia insignis* (Figure 4B). Due to the small sample size the correlation for the mode of failure in the branch could not be tested statistically in *F. insignis*. It is noticeable in *D. reflexa* that smaller branches tend to crack in the branch and that less force is needed than for the other modes of failure (Table 2). In *D. reflexa* the maximal force found for the failure mode ‘failure in the branch’ is significantly smaller than for the other modes of failure (Table 1). Maximal forces found in branches with diameters ranging from 3.5 to 5.4 mm are significantly lower in *D. reflexa* (94.30 ± 48.53 N, Table 2) than in branches of *F. insignis* with the same diameter range (199.62 ± 117.37 N, Table 2).

**Table 1:** Statistical analysis of biomechanical and morphological data of *Dracaena reflexa* and *Freycinetia insignis*^a^*.*

	*Dracaena reflexa*								*Freycinetia insignis*				
	
	failure mode	n	Nor- mality test	KW^b^	Dunn's^c^		Correlation^c^		failure mode	n	Nor- mality test	Correlation^d^	
		
					sickle	stem	failure mode	All				failure mode	All

Maximal force *F*_max_ [kN]	branch	7	-	+	+	+	↑	↑	branch	3	+	/	↑
				
	sickle	13				-	↑		flat	16		↑	
			
	stem	11					↑		\				

Fracture toughness until *F*_max_ [kJm^−2^]	branch	7	-	-	/		-	↑	branch	3	+	/	-
			
	sickle	13					-		flat	16		↑	
		
	stem	11					-		\				

Fracture toughness until failure [kJm^−2^]	branch	7	-	-	/		-	↑	branch	3	+	/	-
			
	sickle	13					-		flat	16		↑	
		
	stem	11					-		\				

Stress at failure [MPa]	branch	7	-	-	/		-	↓	branch	3	+	/	-
			
	sickle	13					-		flat	16		-	
		
	stem	11					-		\				

Strain at failure [/]	branch	7	-	+	-	+	↑	↓	branch	3	-	/	-
			
	sickle	13				-	-		flat	16		-	
		
	stem	11					-		\				


^a^Normality test, Kruskal–Wallis test or Dunn’s test with 5% level of significance (+) or below (-). The Pearson product moment test or the Spearman rank correlation test indicate whether the increase of biomechanical data (e.g., maximal force *F*_max_) is correlated with an increase (↑) or decrease (↓) of the branch diameter or whether there is no statistically significant correlation (-). ^b^The Kruskal–Wallis test is a non-parametric method for testing equality of population medians among groups. ^c^The Dunn's test was used. ^d^The parametric Pearson product moment test was used.

**Table 2:** Morphological and biomechanical data of *Dracaena reflexa* and *Freycinetia insignis*^a^.

	*Dracaena reflexa* (branch diameter 3.52–5.35 mm)						*Freycinetia insignis* (branch diameter 3.52–5.35 mm)						Stat. sign. differ- ence
	
	failure mode	n	mean value	s.d.	mean value all	s.d. all	failure mode	n	mean value	s.d.	mean value all	s.d. all	

Maximal force *F*_max_ [kN]	branch	6	56.06	37.41	94.30	48.53	branch	1	89.02	/	199.62	117.37	+^b^
	
	sickle	5	118.76	42.65			flat	11	209.67	117.55			
	
	stem	3	130.02	19.39			\						

Fracture toughness until *F*_max_ [kN]	branch	6	10.41	4.91	10.53	4.92	branch	1	17.25	/	14.93	9.31	-^b^
	
	sickle	5	9.42	3.08			flat	11	14.72	9.74			
	
	stem	3	12.62	8.27			\						

Fracture toughness until failure [kN]	branch	6	10.74	5.10	11.01	5.15	branch	1	17.74	/	15.54	9.56	-^c^
	
	sickle	5	10.09	3.15			flat	11	15.34	10.01			
	
	stem	3	13.08	8.89			\						

Stress at failure [MPa]	branch	6	30.40	11.38	27.86	11.17	branch	1	42.95	/	50.11	14.26	+^c^
	
	sickle	5	26.54	10.93			flat	11	50.76	14.77			
	
	stem	3	24.96	14.57			\						

Strain at failure [/]	branch	6	0.85	0.58	0.58	0.48	branch	1	0.82	/	0.31	0.19	-^b^
	
	sickle	5	0.47	0.33			flat	11	0.27	0.11			
	
	stem	3	0.21	0.11			\						


^a^The significance of the differences in *D. reflexa* and *F. insignis* was calculated with the Kruskal–Wallis test or One Way Analysis of Variance with a 5% level of significance (+) or below (-). ^b^Data tested with Kruskal–Wallis One Way Analysis of Variance on Ranks. ^c^Data tested with One Way Analysis of variance.

#### Fracture toughness until *F*_max_ [kJm^−2^]

**Figure 5:**
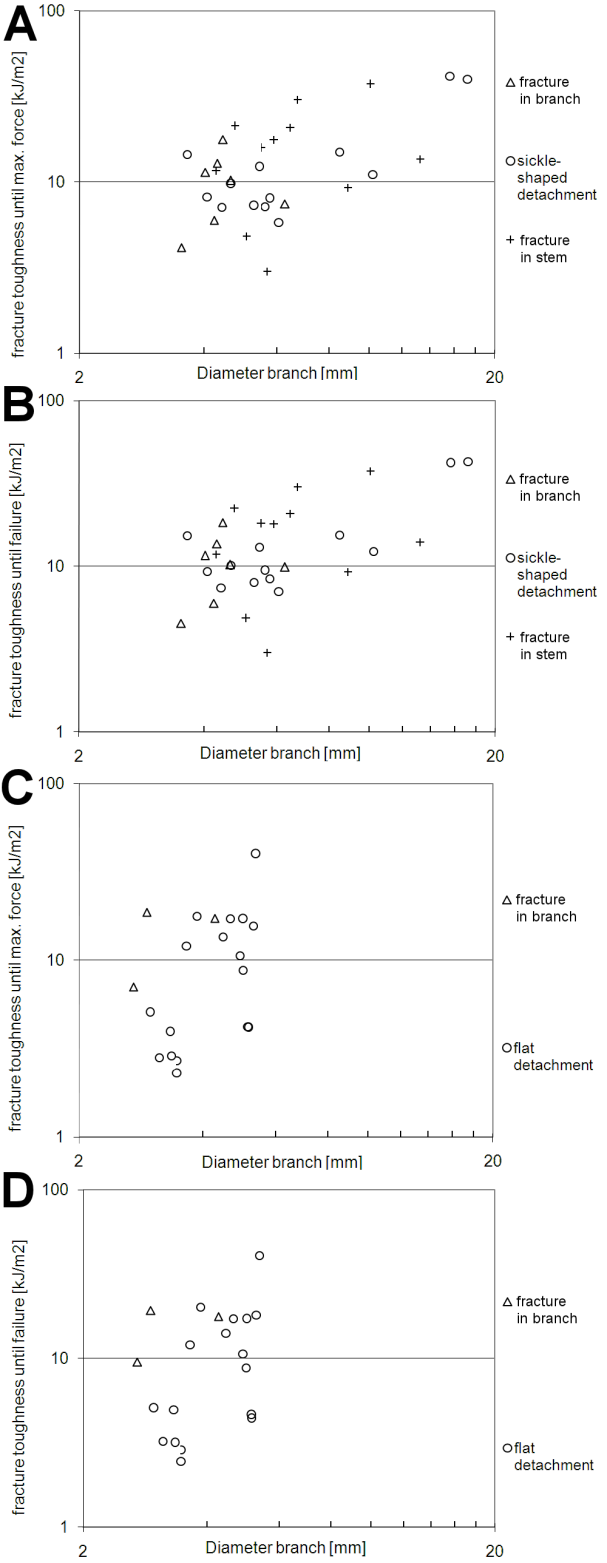
Bivariate, double logarithmic plot of fracture toughness until maximal force vs diameter of lateral branches (*d*_1_ or *d*_2_ depending on the mode of fracture (see ‘Experimental’ section) in *Dracaena reflexa* (A) and *Freycinetia insignis* (C). Bivariate, double logarithmic plot of fracture toughness until failure vs diameter of lateral branches (*d*_1_ or *d*_2_ depending on the mode of fracture (see ‘Experimental’ section) in *Dracaena reflexa* (B) and *Freycinetia insignis* (D).

The increase of the fracture toughness until *F*_max_ with increasing diameter of the lateral branches is statistically significant only for the pooled data of all fracture modes in *D. reflexa* (Figure 5A, Table 1). A similar but not statistically significant trend can also be observed in *F. insignis* (Figure 5C, Table 1). No statistically significant difference exists between the different failure modes in *D. reflexa*. Fracture toughness until *F*_max_ found in branches with diameters from 3.5 to 5.4 mm is not significantly different in *D. reflexa* (10.53 ± 4.92 kJm^−2^, Table 2) compared to *F. insignis* (14.93 ± 9.31 kJm^−2^, Table 2).

#### Fracture toughness until failure [kJm^−2^]

The increase of the fracture toughness until failure also increases statistically significant with increasing diameter of the lateral branches for the pooled data of all fracture modes in *D. reflexa* (Figure 5B, Table 1). A similar but not statistically significant trend can also be observed in *F. insignis* (Figure 5D, Table 1). No statistically significant difference was found between the different failure modes in *D. reflexa*. Fracture toughness until failure found in branches with diameters from 3.5 to 5.4 mm is not significantly different in *D. reflexa* (11.01 ± 5.15 kJm^−2^, Table 2) compared to *F. insignis* (15.54 ± 9.56 kJm^−2^, Table 2).

#### Stress at failure [MPa]

**Figure 6:**
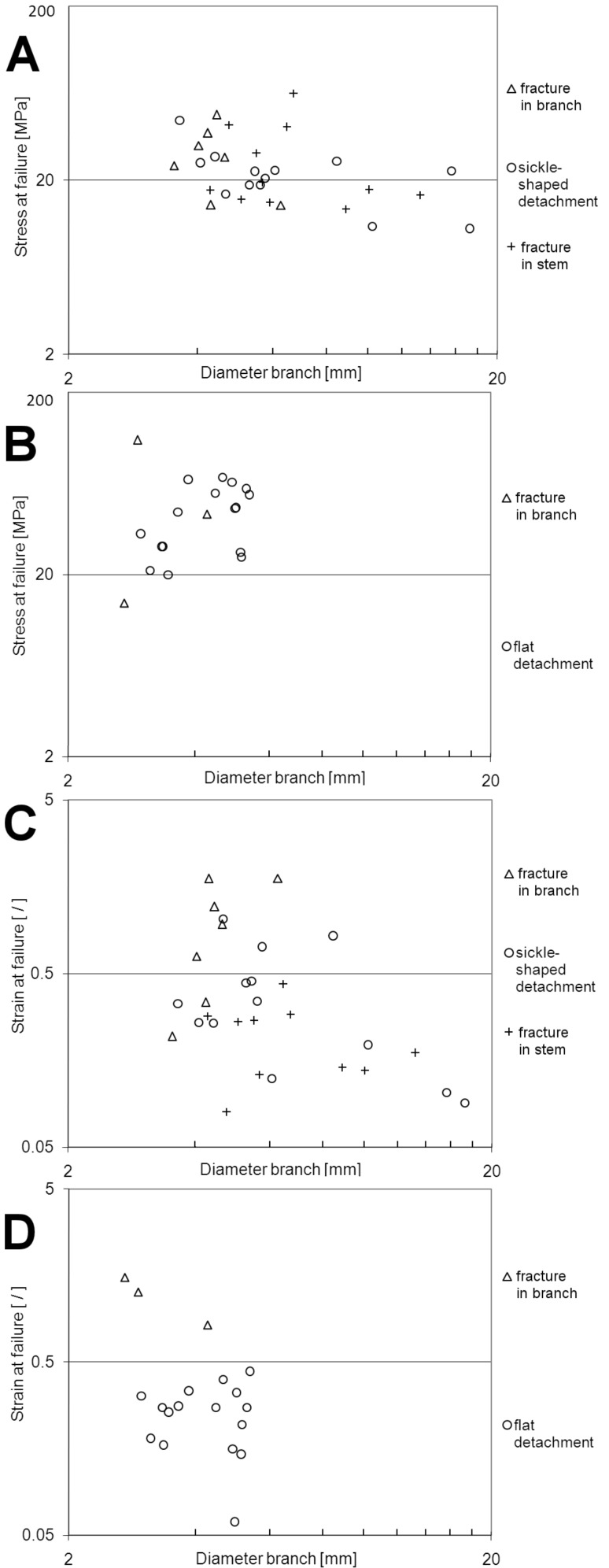
Bivariate, double logarithmic plot of stress at failure vs diameter of lateral branches (*d*_1_ or *d*_2_ depending on the mode of fracture (see ‘Experimental’ section) in *Dracaena reflexa* (A) and *Freycinetia insignis* (B). Bivariate, double logarithmic plot of strain at failure vs diameter of lateral branches (*d*_1_ or *d*_2_ depending on the mode of fracture (see ‘Experimental’ section) in *Dracaena reflexa* (C) and *Freycinetia insignis* (D).

The data do not show a statistical difference between the different failure modes in *D. reflexa* (Figure 6A, Table 1). A statistically significant trend only exists for the decrease of stress at failure with increasing diameter in the pooled data of all fracture modes in *D. reflexa* (Figure 6A, Table 1). No correlation between stress at failure and diameter of the lateral branches could be observed in *F. insignis* (Figure 6B, Table 1). In branches with diameters from 3.5 to 5.4 mm stress at failure is significantly higher in *F. insignis* (50.11 ± 14.26 MPa, Table 2) than in *D. reflexa* (27.86 ± 11.17 MPa, Table 2).

#### Strain at failure [ / ]

In *D. reflexa*, strain at failure is significantly higher for the mode ‘failure in the branch’ than in the mode ‘failure in the stem’ (Figure 6C, Table 1). The failure mode ‘sickle-shaped detachment’ does not differ from both other failure modes. The decrease of strain at failure with increasing branch diameters is significant for the pooled data of all fracture modes in *D. reflexa* (Figure 6C, Table 1). For the mode ‘failure in the branch’ a significant increase of strain at failure with increasing diameter was found. No correlation between strain at failure and diameter of the lateral branches could be observed in *F. insignis* (Figure 6D, Table 1). In branches with diameters from 3.5 to 5.4 mm, no significant difference for the strain at failure could be found between *D. reflexa* (0.58 ± 0.48, Table 2 and *F. insignis* (0.31 ± 0.19, Table 2).

## Discussion

### Morphology and biomechanics

The monocotyledonous mode of branching has important implications for the morphology and anatomy in *Dracaena reflexa* and *Freycinetia insignis*. It determines the shape of the branch–stem-junctions, the arrangement and course of the fibrous bundles in the main stem and in the lateral branches and their interconnection. The mode of branching again depends on the apical or distal position of the branching on the stem [2]. Due to this, a certain variability of the attachment modes exists, which may account for the relatively large scatter of the biomechanical data (Figures 4–6). It can be assumed that the ability to build secondary tissues has a high impact on growth form and biomechanics. While the attachment modes of *D. reflexa* and *F. insignis* are generally similar in that the lateral branches clasp around the main stem (Figure 2), the distinct separation (in some parts by a layer of parenchymatous tissues) of lateral branches from the main stem found in *F. insignis* contrasts with the gradual transition between fibrous bundles secondarily formed in the main stem and the lateral branches observed in *D. reflexa* (Figure 2B,D). Furthermore, due to the missing ability for anomalous secondary growth, *F. insignis* is not able to form secondary tissues [26] including fibrous bundles. Therefore, this species cannot (or only to a very limited extent) ‘react’ adaptively to an increase in loads acting on stem, branch and branch–stem-junction when the length of the branch increases. It can therefore be assumed that the branch–stem-junctions in *F. insignis* are configured to carry high loads from the very start of their development, i.e., when these interconnections are formed by primary establishment growth. This could explain why *F. insignis* is able to carry higher loads in small branches compared to *D. reflexa* (Table 2)*.*

#### Qualitative biomechanics: Failure modes

In *D. reflexa*, the fibrous bundles that are connected to the lateral branches clasp around the main stem resulting in a sickle-shaped structure (Figure 2B,D, see Supporting Information File 1). This accounts for the high number of sickle-shaped failures in which the outer fibrous bundles remain attached to the lateral branches and are detached from the main stem (Figure 3B) following the stem clasping arrangement of the fibrous bundles of the branch (Figure 2B,D, see Supporting Information File 1). Failure in the branch occurs mainly in relatively young, possibly not fully lignified branches (see below). Failure in the stem results in longitudinal cracks which very quickly reach a critical length and lead to failure of the main stem. This failure mode is the most disadvantageous one for the plant and can be lethal as the mechanical stability of the stem is drastically reduced, water and assimilate transport is seriously disturbed and germs can easily access and infect the large wound surface of the plant. It remains unclear whether and to what extent this failure mode may occur due to an experimental artefact that occurs in spite of clamping the apical cut off part of the stem circumferentially with a clip collar to avoid longitudinal splitting.

In *F. insignis*, the fibrous bundles are arranged in a different pattern with no gradual transition between the fibrous bundles in the main stem and the fibrous bundles in the lateral branches as found in *D. reflexa*. The fibrous bundles in stem and branch in *F. insignis* are anatomically strictly separated (Figure 2A,C), and the detachment modes mirror this arrangement. *F. insignis* typically (in 84% of failures) shows a detachment of the fibrous bundles of the lateral branches from those of the main stem resulting in a relatively flat fracture surface. In some cases (in 16% of failures) branch–stem-junctions with low diameters of lateral branches (under 1 mm) fail in the branch. It can be speculated that these very young branches are not yet fully lignified and therefore structurally weaker than thicker ones. Very young stems in *F. insignis* have fully lignified fibrous bundles but they lack a somewhat more densely packed zone of lignified fibrous bundles at the transition between stem and branch, also the ground tissue appears to be less lignified than the ground tissue in older axes.

#### (Semi-)Quantitative biomechanics

One of the most prominent results is that the values for maximal force and for stress at failure are significantly higher in *Freycinetia insignis* than in *Dracaena reflexa*. In fact all tested mechanical parameters are higher in *F. insignis*, even if not significantly. A possible reason for this may be the lack of anomalous secondary growth in *F. insignis* which is thereby restricted to develop only comparatively thin main stems and lateral branches throughout its entire ontogenetic trajectory. It can be argued that these ‘thin branchings’ in *F. insignis* with diameters of lateral branches between 3.52 and 5.35 mm are fully mature and are able to take up higher loads than branches in *D. reflexa* with the same diameter. In contrast to *F. insignis*, the same diameters are relatively small for lateral branches of *D. reflexa* so that the fibrous bundles may not yet have reached a fully lignified state. This is indicated by staining experiments for lignin in which young axes of *Dracaena reflexa* (3–5 mm) are less stained than older axes (above 7 mm).

#### Comparison with data of branchings of dicotyledonous trees

No comparative data for maximal force or fracture toughness measured with a similar setup could be found for dicotyledonous trees in the literature. However, the maximal forces measured for branching of *D. reflexa* and *F. insignis* are intuitively high with a mean value of 199.62 ± 117.37 N and maximum values of up to 0.3 kN for *F. insignis* in a branch no thicker than 5mm (in larger stems of *Dracaena reflexa* with a branch diameter of 20 mm, the maximal force exceeded 10 kN, data not shown here). Values for fracture toughness can be also considered to be high as the maximal forces are high and large displacements are generated before the branches break off.

Values for stress at failure are comparable to values found for dicotyledonous trees, [4] reported for different species of willows with an identical experimental setup and comparable branch diameters between 2 and 6 mm, mean values for stress at failure ranging from 18 to 57 MPa.

On the other hand, the values for strain at failure are much higher than the values reported for dicotyledonous trees [4] found for different species of willows with an identical experimental setup with mean values for the strain at failure that ranged from 6% to 11%. These values are very much lower than the 58% strain at failure found in *D. reflexa* and the 31% strain at failure found in *F. insignis*.

Based on the structural data presented, one may hypothesize that one reason for the high values of strain at failure found in the two arborescent monocotyledon species is the special stem architecture with loosely interconnected fibrous bundles in a rather flexible parenchymatous ground tissue. Insertion of the fibrous bundles and gradual connection with the ground tissue as well as the intertwining arrangement of the fibrous bundles leads to a structure which allows for a high load bearing capacity combined with high fracture toughness and very high strains at failure.

### Outlook: biomimetic potential and implementation

**Figure 7:**
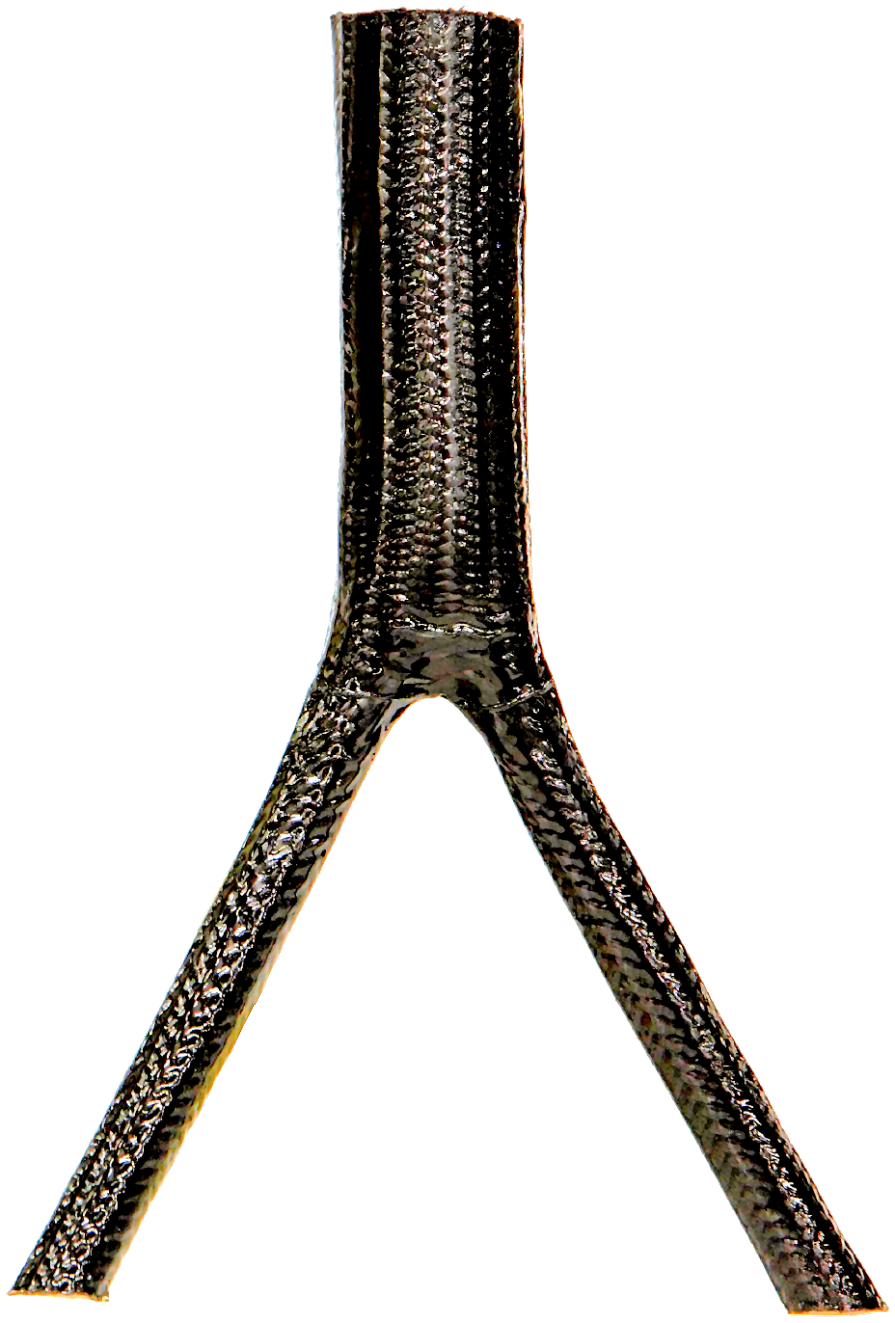
Prototype of biomimetically optimised, braided branched fibre reinforced technical compound structure © ITV Denkendorf.

These mechanical properties make the arborescent monocotyledons studied well suited as concept generators for technical implementations in branched fibre-reinforced compound structures such as axel-carriers and frames in automotive engineering and aerospace as well as ramified supporting structures in architecture. The high potential of arborescent monocotyledons is currently being assessed in a joint project whose members include the Plant Biomechanics Group Freiburg, the Institute for Textile Technology and Process Engineering Denkendorf, the Institute of Lightweight Structures and Polymer Technology of the TU Dresden as well as the Botanical Garden of the TU Dresden [27–31]. In this project the hierarchical organisation of branch–stem-junctions of arborescent monocotyledons and columnar cacti is analysed and different biomechanical tests are performed on these plants in order to determine the mechanical parameters of stems, branches, branch–stem-junctions and the different constituent tissues in different directions. This allows for simulation of hierarchical structure and mechanical behaviour of the biological structures and thereby to identify the underlying principles of mechanical optimisation. Some of these principles have already been quantitatively understood, abstracted and first prototypes have been produced that already incorporate different structural and mechanical optimisations (Figure 7).

## Experimental

### Sampling of *Dracaena reflexa* and *Freycinetia insignis*

The biomechanical properties of 127 branch–stem-junctions of *D. reflexa* and of 37 branch–stem-junctions of *F. insignis* were analysed. Of these, 31 ramifications of *D. reflexa* and 19 of *F. insignis* could be retained for the statistical analysis (Table 1).

The specimens used for this study originated from commercial garden centres (*D. reflexa*) and from the Botanic Garden of Freiburg (*F. insignis*). These plants were chosen as they show a relatively high number of branchings and a lignified branching region with anomalous secondary growth (*D. reflexa,* Figures 1E,F, 2B,D), or without anomalous secondary growth (*F. insignis,* Figures 1G, 2A,C). For the measurements in *D. reflexa* two varieties of *Dracaena* were used: ‘Song of India’ and ‘Song of Jamaica’. These varieties only differ by the form and colour patterns of their leaves while morphology, anatomy and the biomechanical behaviour showed neither apparent nor statistically significant differences. Consequently, the values of these two varieties are pooled and presented as ‘*D. reflexa*’.

### Anatomy and morphology

Thin and semi-thin sections for anatomical analysis were obtained via microtome sectioning and staining with the Fuchsin*-*Chrysoidin*-*Astrablue staining method according to Etzold [32] (Figure 2). Additionally, the three-dimensional arrangement and the course of the fibrous bundles was analysed by superimposing photographs of serial sections showing the two-dimensional arrangement of the fibrous bundles one upon the other in order to obtain a stacked sequence (following a similar technique as the one described in [33]) that could be digitized and visualised (see Supporting Information File 1).

### Morphometric measurements

**Figure 8:**
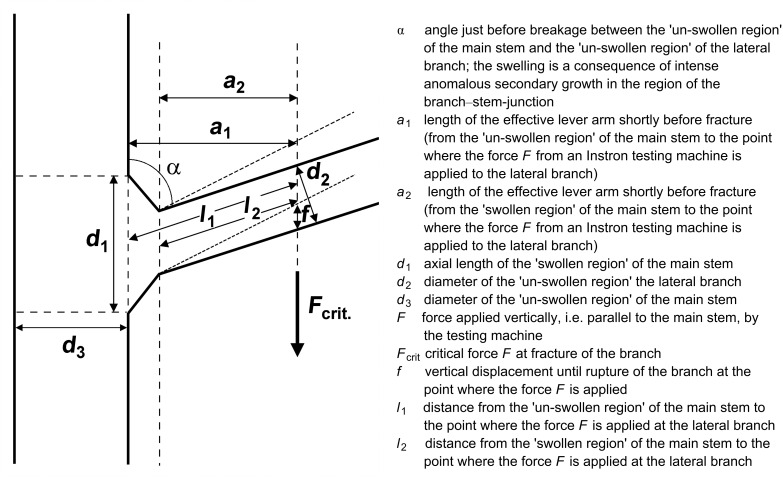
Breaking experiments. Schematic drawing of the geometry and parameters used for calculations. The dashed lines show a lateral branch before bending, the solid lines show a main stem and a lateral branch shortly before fracture.

Different lengths, diameters, and angles were measured in the main stems and lateral branches of the selected arborescent monocotyledons (Figure 8). These measurements were also carried out in the ‘swollen part’ where the lateral branches are connected to the main axis. This region has developed by intense ‘anomalous secondary growth’, and it can be assumed that it is of (high) mechanical importance for the biomechanical performance of the branch stem-junctions.

### Experimental setup

**Figure 9:**
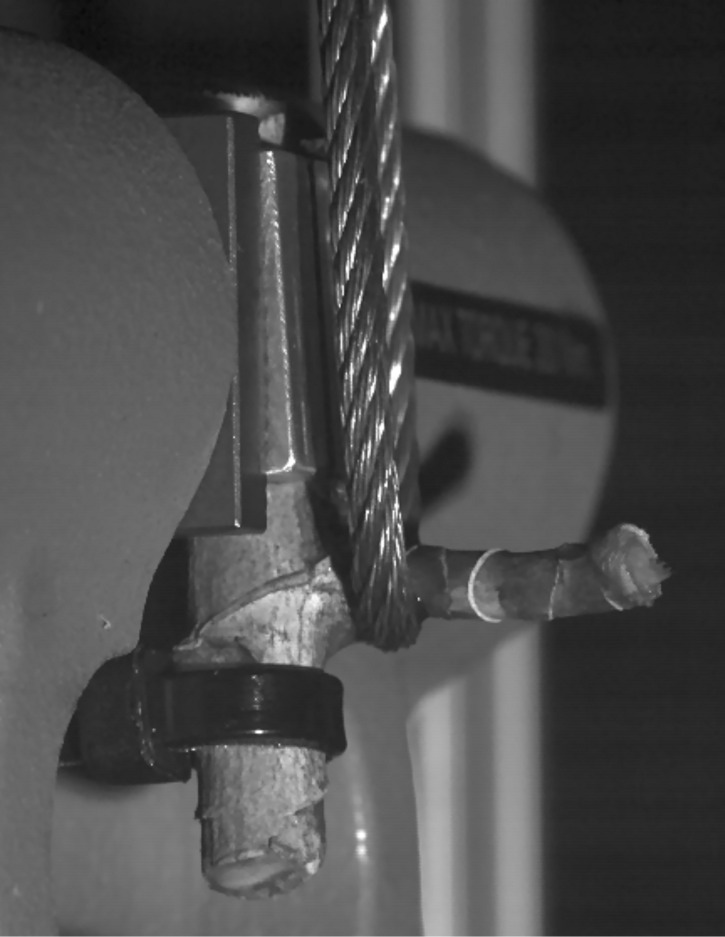
Breaking experiments. A critical force *F*_crit_ is applied to a branch of *Dracaena reflexa* by means of a steel cable connected to an Instron testing device. For technical reasons (direction of movement of the cross-head) the branch–stem-junctions are oriented ‘upside-down’ with regard to the original orientation in the plant.

The upper part of the cut off main axis was tightly embraced by a cable strap (Figure 9). This gripping helps to avoid longitudinal fractures in the main axis that could result as artefacts from the proximity of the branching region to the point where the main axis is cut off. A steel cable connected to the testing machine was placed around the branch (Figure 9). When the cross-head of the testing machine moves upwards, the steel cable tears off the lateral branch from the main axis (due to the upward movement of the cross-head, the branch–stem-junctions were oriented ‘upside-down’ in the testing device, see Figure 9). The speed of the cross-head was set to 100 mm/min. The experiments were recorded with a high speed camera at 1000 fps in order to determine the vertical displacement until rupture of the branch at the point where the force *F* was applied (‘*f*’ in Figure 8). The design of the setup as well as the biomechanical calculations were carried out according to the methods described in detail in [4].

### Biomechanical calculations

Our experiment showed that three distinct modes of failure exist when tearing off lateral branches in *Dracaena reflexa* (Figure 3). When the rupture occurred in the ‘un-swollen region’ of the lateral branches, the parameters *a*_2_, *l*_2_ and *d*_2_ (Figure 8) were used for the calculations. When the branch–stem-junctions mechanically failed either in the ‘swollen region’ between main stem and lateral branch or in the main stem itself, *a*_1_, *l*_1_ and *d*_1_ were used; *r*_1_ and *r*_2_ are the radius of the main stem and the lateral branch, respectively, where:


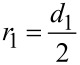
 and 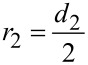 [m]

**Axial second moment of area (I),** assuming a circular cross-section of main stem and lateral branch which holds true in good approximation for the investigated species


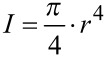
 [m^4^], *r* = *r*_1_ or *r*_2_ depending on the mode of fracture

**Bending moment at fracture (BM)**


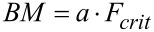
 [Nm]

*a* is the length of the effective lever arm shortly before fracture; *a*_1_ or *a*_2_ are used depending on the mode of fracture, 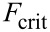 is the critical force at rupture

**Stress at rupture (σ)**


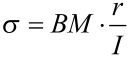
 [Nm^−2^], *r* = *r*_1_ or *r*_2_ depending on the mode of fracture

**Strain at rupture (ε)**


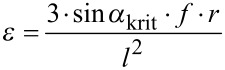
 [ / ]

*f* is the vertical displacement until rupture of the branch at the point where the force *F* is applied to the lateral branch

*l* is the length between the point where the force is applied to the lateral branch and the position of rupture in the main stem or the lateral branch, *l* = *l*_1_ or *l*_2_ depending on the mode of fracture


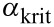
 is the angle between the lateral branch and main stem at the moment of rupture

**Tensile energy absorption [kJ]**

**Figure 10:**
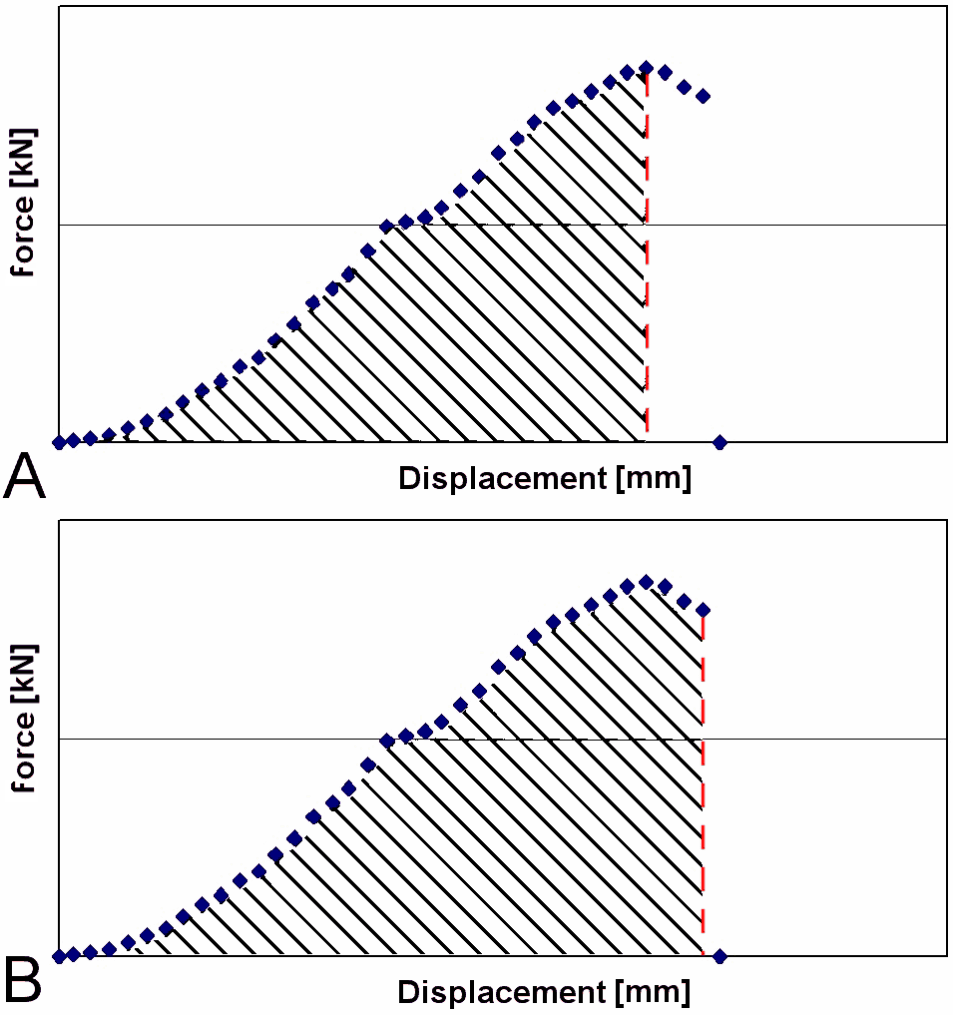
Breaking experiments. Exemplary force-displacement diagrams showing maximal force and displacement at maximum force (A) as well as force at fracture and displacement at fracture (B). The hatched areas were used for calculating fracture toughness until maximum force (A) and work of fracture until failure (B).

The tensile energy absorption up to the maximal force or up to the force at fracture was assessed by calculating the integral under the force-displacement-curve in the force-displacement diagrams (Figure 10).

**Fracture toughness [kJm^−2^]**

Fracture toughness was calculated by dividing the tensile energy absorption by the cross-sectional area calculated for the main stem of the lateral branch by using *d*_1_ or *d*_2_ depending on the mode of fracture (see above).

### Statistical calculations

The software Sigmastat (Version 3.1) by Systat Software Inc. was used. Not all recorded data were normally distributed (Table 1). Therefore parametric tests (normal distribution) or non-parametric tests (no normal distribution) were used for calculating statistical significance of parameter correlation or difference amongst groups (Table 1).

- The (parametric) ‘One Way Analysis of Variance’ or the (non-parametric) ‘Kruskal–Wallis One Way Analysis of Variance on Ranks’ was used:

1. to verify whether the three groups of observed failure modes (in the branch, sickle-shaped, in the stem) for *D. reflexa* were statistically different as to the mechanical parameters calculated (p < 0,05, Table 1).
2. to analyse whether the biomechanical data for *D. reflexa* differed from the data of *F. insignis* (Table 2)*.* Only specimens of *D. reflexa* that were found to be in the same range of branch diameters as *F. insignis* (3.5–5.4 mm) were used for this comparison (n = 14 for *D. reflexa*, n = 12 for *F. insignis*; Table 2).

- The Pearson Product moment correlation test was used to calculate whether there was a statistically significant correlation between tested mechanical parameters (e.g., maximum force) and the diameter of the branch (Table 1).

- No statistical significance of differences between the failure modes in *F. insignis* could be calculated, as the number of specimens in one failure mode was too low (n = 3, Table 1) in order to make a reasonable statistical evaluation.

## Supporting Information

File 1 Three-dimensional arrangement and course of fibrous bundles in a branch–stem-junction of *Dracaena reflexa.*
